# Altered virulence of Highly Pathogenic Avian Influenza (HPAI) H5N8 reassortant viruses in mammalian models

**DOI:** 10.1080/21505594.2017.1366408

**Published:** 2017-09-21

**Authors:** Su-Jin Park, Eun-Ha Kim, Hyeok-Il Kwon, Min-Suk Song, Se Mi Kim, Young-Il Kim, Young-Jae Si, In-Won Lee, Hiep Dinh Nguyen, Ok Sarah Shin, Chul-Joong Kim, Young Ki Choi

**Affiliations:** a Department of Microbiology, College of Medicine and Medical Research Institute, Chungbuk National University, Cheongju, Republic of Korea; b Zoonotic Infectious Diseases Research Center, Chungbuk National University, Cheongju, Korea; c Brain Korea 21 Plus for Biomedical Science, College of Medicine, Korea University, Seoul, Republic of Korea; d College of Veterinary Medicine, Chungnam National University, Daejeon, Republic of Korea

**Keywords:** H5N8, HPAI influenza virus, PB2, reassortment, virulence

## Abstract

Recently identified highly pathogenic avian influenza (HPAI) H5N8 viruses (clade 2.3.4.4) are relatively low to moderately pathogenic in mammalian hosts compared with HPAI H5N1 viruses. In this study, we generated reassortant viruses comprised of A/MD/Korea/W452/2014(H5N8) with substitution of individual genes from A/EM/Korea/W149/2006(H5N1) to understand the contribution of each viral gene to virulence in mammals. Substituting the PB2 gene segment or the NA gene segment of the H5N8 virus by that from the H5N1 virus resulted in significantly enhanced pathogenicity compared with the parental H5N8 virus in mice. Of note, substitution of the PB2 gene segment of the H5N8 virus by that from the H5N1 virus resulted in a 1000-fold increase in virulence for mice compared with the parental virus (MLD_50_ decreased from 10^5.8^ to 10^2.5^ EID_50_). Further, the W452_W149PB2_ virus also induced the highest virus titers in lungs at all time points and the highest levels of inflammatory cytokine responses among all viruses tested. This high virulence phenotype was also confirmed by high viral titers in the respiratory tracts of infected ferrets. Further, a mini-genome assay revealed that W452_W149PB2_ has significantly increased polymerase activity (*p* < 0.001). Taken together, our study demonstrates that a single gene substitution from other avian influenza viruses can alter the pathogenicity of recent H5N8 viruses, and therefore emphasizes the need for intensive monitoring of reassortment events among co-circulating avian and mammalian viruses.

## Introduction

Since the mid-1990s highly pathogenic avian influenza (HPAI) A(H5N1) viruses have ravaged domestic poultry in Asia. The unprecedented spread of these viruses toward Africa and Europe since 2003 has given rise to at least 10 distinct phylogenetic clades based on the antigenicity of their hemagglutinin (HA) genes.^1^ Endemicity of highly pathogenic A(H5N1) influenza A viruses in wild birds and poultry has caused over 850 human infections and continues to pose a public health risk.^2^ In Asia the co-circulation of these viruses with other prevailing influenza viruses has led to the generation of novel reassortant H5Nx strains with unique gene constellations.^3-5^

A newly emerged, HPAI A(H5N8) virus caused poultry outbreaks in the Republic of Korea in mid-January 2014.^6^ Despite control measures on A(H5N8)-infected farms, the virus still managed to spread widely in Korea, resulting in the culling of more than 19 million poultry birds within a short period of time.^7^ Even though all segments of the 2014 HPAI A(H5N8) virus were derived from strains that are resident in Eurasian migratory wild birds, there were no segment originating from A(H9N2) such as is typically seen with emergence of novel human-infecting A(H7N9) or A(H10N8) avian viruses in China.^8,9^ These HPAI H5N8 viruses have spread to China, Japan, Europe and even into North America, where they were detected in domestic and wild birds.^10-13^ In addition, genetic reassortment with circulating avian influenza viruses has resulted in novel HPAI viruses, including H5N1, H5N2, H5N6, and H5N8 subtypes^14,15^ Recently, novel H5N6 and H5N8 viruses (reassortant viruses from at least 3 different genetic origins) were identified in Eurasia including in South Korea.^16-19^ These reports suggested that active reassortments commonly occur in nature and are a source for novel HPAI viruses.

Although HPAI H5N8 is highly pathogenic in chickens, it has thus far been extremely poor at inducing remarkable clinical illness in domestic ducks and ferrets, which may allow establishment of the virus in these hosts.^20^ Furthermore, the virus is only moderately pathogenic in mice and has limited tissue tropism relative to that of previous HPAI H5N1 viruses. Although these findings minimize the concern that HPAI A(H5N8) viruses with their present phenotypes would cause human infections, there are indications that the virus may have the potential to become endemic in domestic poultry, which could alter the genetic evolution of pre-existing strains. For example, H5N6 viruses were recently isolated from various provinces in China where they caused human infections. Strikingly, of 16 human H5N6 infection cases, 6 patients eventually died suggesting that clade 2.3.4.4 H5 viruses from avian species could directly infect humans and cause significant concern for public health^21,22^

Although most HPAI H5N1 viruses cause severe clinical symptoms and high mortality in experiment animal models including mice, the recent H5N8 viruses showed only moderate virulence in mice, similar to the 2009 pH1N1 viruses (both viruses show an MLD_50_ of 10^5.8^ EID_50_).^23^ Detailed studies of virulence factors and the pathogenic potential of novel H5N8 and its reassortants arising from exchange with preexisting H5 viruses are currently limited. Given the threat of the worldwide spread of the H5N8 virus and its co-circulation with previous HPAI H5N1 viruses, we investigated the HPAI H5N8 virulence factors in detail by comparing them with recent HPAI H5N1 (A/EM/Korea/W149/2006, clade 2.2). Further, we evaluated the pathogenic potential of possible H5N8 reassortants in mouse and ferret models.

## Results

### Generation of H5N8-H5N1 reassortant viruses

To investigate whether the biologic features of low to moderately virulent H5N8 (W452) virus were altered in mammals following reassortment events with the HPAI H5N1 (W149. Clade 2.2) virus, variants were plaque purified in Madin-Darby canine kidney (MDCK) cells following co-infection and were subsequently genotyped by RT-PCR using custom-designed gene-specific primers. Reassortants were then confirmed by full-length sequencing. The resulting plaque-purified reassortant viruses and selected single gene-reassortants used in this study are listed in Table 1. Reassortant viruses were named according to the origin of their exchanged genes. For example, W452_W149PB2_ denotes a reassortant bearing the PB2 of W149 with the rest of its genes from W452. Hence, a total of 11 viruses, including parental H5N8 and H5N1 viruses as well as a W149_W452PB2_ reassortant, were used in this study.

**Table 1.** Growth properties of viruses *in vitro* and virulence in mice.

	Virus stock (log_10_EID_50_/ml)	MLD_50_^b^(log_10_EID_50_)
**^a^W149**	9.5	3.3
**^a^W452**	8.8	5.8
W452_W149PB2_	9.5	2.5
W452_W149PB1_	8	>6.5
W452_W149PA_	9.8	5.8
W452_W149HA_	8.8	5.7
W452_W149NP_	8.8	>6.5
W452_W149NA_	7	4.8
W452_W149M_	8.8	>6.5
W452_W149NS_	8.8	>6.5
W149_W452PB2_	9.5	4.5

Notes.

a Reassortant virus names were assigned according to the origin of the exchanged genes (e.g., 149 PB2 denotes a reassortant bearing the PB2 of 149 and the rest of its genes from 452. Parental strains are in **boldface** type.

b Determined by inoculating mice with 10^2.5^ to 10^6.5^ EID_50_ of virus and expressed as the log_10_ EID_50_ required to yield 1 MLD_50_.

To make the virus stock, each virus was propagated into 10-day-old embryonated chicken eggs and titered via the 50% egg infectious dose (EID_50_) end point. Each reassortant virus containing the PB1, HA, NP, M, or NS gene of W452 showed similar growth properties to the parental W452 virus in embryonated chicken eggs (Table 1). Insertion of the PB2 or PA genes from W149 into the W452 backbone increased virus titers to more than 10^9.5^ EID_50_/ml, while the NA gene (N1) substitution virus (W452_W149NA_) exhibited reduced viral titers compared with the parental W452 virus (10^7^ vs. 10^8.8^ EID_50_/ml).

### Substitution of the PB2 gene renders the low-pathogenic H5N8 virus highly pathogenic in mice

**Figure 1.**
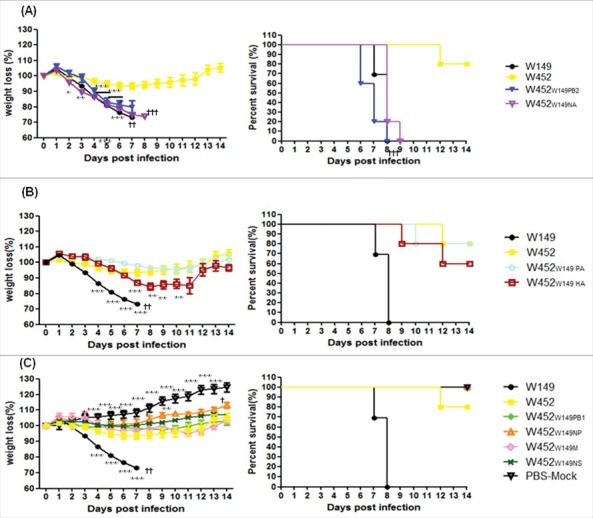
Pathogenicity of recombinant W452 reassortant viruses carrying W149 segments in mice. Groups of mice (n = 10 per group) were intranasally inoculated with 10^5.5^ EID_50_ of each virus. All groups were observed for morbidity and mortality for 14 d. Any mouse that lost >25% of initial body weight was killed. The percentage of mean weight loss (left panel) and survival (right panel) are shown. Viruses were classified according to high, moderate, and low pathogenicity (A) highly pathogenic group, (B) moderately pathogenic group, and (C) low-pathogenic group. The results were confirmed by 2 independent experiments. Statistically significance weight loss was determined by one-way ANOVA and subsequent Dunnett's test and is marked with asterisks (between W452 and other viruses) or daggers (across time points). Further, the Mantel Cox method was used to assess survival. (* or † indicates p < 0.05, **or †† indicates ** p < 0.01, and *** or ††† indicates *p < 0.001*).

To verify whether the different *in vitro* growth properties of these viruses correspond with the pathogenic properties of H5N8 (W452) in mammalian hosts, their 50% mouse lethal doses were determined (Table 1). In addition, groups of mice were inoculated with 10^5.5^ EID_50_ of each of the single-gene reassortants or parental virus, and changes in weight and survival rates were observed. The mice infected with the wild-type H5N8 virus showed moderate pathogenic features, with 80% survival within 14 d post infection (dpi) (MLD_50_; 10^5.8^ EID_50_), while all mice infected with parental H5N1 (W149) virus succumbed to death or were killed due to more than 25% weight loss and/or severe clinical signs within 8 dpi (MLD_50_; 10^3.3^ EID_50_) (Table 1 and Fig. 1). For the reassortants, virulence was categorized into one of 3 groups including (i) highly pathogenic group (Fig. 1A), (ii) moderately pathogenic group (Fig. 1B) and (iii) low pathogenic group (Fig. 1C). To compare reassortants with wild type viruses, we additionally inserted mouse weight loss and survival data of W149 and W452 viruses in grouped Fig. 1A to C. Two viruses (W452_W149PB2_ and W452_W149NA_) were included in the highly pathogenic group. Notably, mice infected with the W452_W149PB2_ virus experienced rapid weight loss of more than 25%, 100% mortality, and an MLD_50_ of 10^2.5^ EID_50_, which is even lower than that of the wild-type H5N1(W149) virus (10^3.3^ EID_50_ of MLD_50_) (Table 1). The W452_W149NA_ virus also had higher pathogenicity (10^4.8^ EID_50_ of MLD_50_) in infected mice, in spite of the fact that it showed a reduced yield in embryonated eggs (10^7.0^ EID_50_/ml). Reassortant viruses including W452_W149PA_ and W452_W149HA_ induced body weight loss and mortality in a manner similar to the wild-type H5N8 virus with moderate virulence (ranging from 10^5.7^ to 10^5.8^ EID_50_ of MLD_50_) (Fig. 1B, Table 1). In contrast, W452_W149PB1,_ W452_W149NP,_ W452_W149M,_ and W452_W149NS_ showed attenuated body weight loss without any mortality (MLD_50_ > 10^6.5^ EID_50_) (Fig. 1C, Table 1). These results clearly demonstrate that in mice the relatively low-pathogenic H5N8 virus can be altered to become a highly virulent virus by single gene reassortment with the recent, highly pathogenic H5N1 virus.

### The PB2 gene from the H5N1 virus expands the tissue tropism of the H5N8 virus in mice

**Figure 2.**
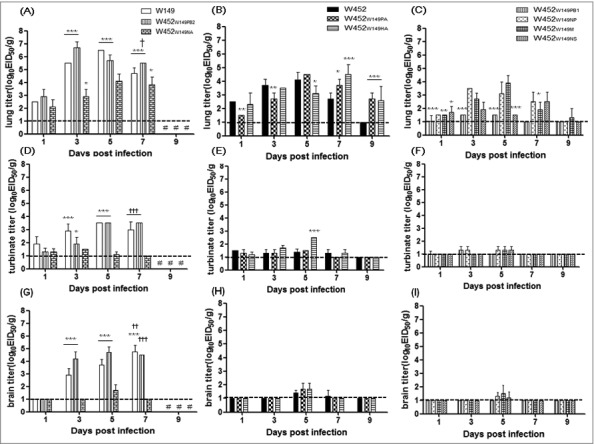
Replication of reassortant viruses in mouse lungs, brains and turbinates. Mice were inoculated with 10^5.5^ EID_50_ and samples (n = 5 per group) were collected on 1, 3, 5, 7, and 9 dpi. A-C, D-F or G-I are viral titers from lungs, turbinate, or brains respectively. (A), (D) and (G) are highly pathogenic, (B), (E) and (H) are moderately pathogenic, (C), (F) and (I) are low-pathogenic viruses. lungs, brains and turbinate titers shown are means ± SD from and titers below the limit are shown as 1 log_10_EID_50_/g (dashed lines). The results were confirmed by 2 independent experiments. #, No samples collected because the mice in this group died. Asterisks indicate statistical significance between W452 and other viruses as determined by one-way ANOVA and subsequent Dunnett's test. Further, daggers indicate the statistical significance across time points. (* or † indicates *p* < 0.05, **or †† indicates ** p < 0.01, and *** or ††† indicates *p* < 0.001).

To further assess if the single gene-reassortant viruses could alter the replication properties of these viruses in mice, virus-infected mouse lungs were harvested at 1, 3, 5, 7, and 9 dpi. Of the highly pathogenic group, the viral titer of the W452_W149PB2_ virus peaked at 3 dpi (10^6.7^ EID_50_/g) and was at least as high as that of the H5N1 (W149) virus (Fig. 2A). Although the W452_W149NA_ virus showed a low (10^4.8^ EID_50_) MLD_50_ (Table 1), the lung viral titer was not significantly different from that of the parental H5N8 virus (Fig. 2A and B). The W452_W149PA_ and W452_W149HA_ viruses showed a delay in the viral titer peak until 5 or 7 dpi and detection of the virus until 9 dpi (Fig. 2B). In addition, virus was detected in the lungs of mice infected with W452_W149M_ virus until 9 dpi (Fig. 2C). In contrast, the other reassortants exhibited relatively decreased viral growth in mouse lung compared with the wild-type H5N8 virus, although each virus persisted up to 5 or 7 dpi in the lung (Fig. 2C).

Multiple organ failure is a typical feature of HPAI H5N1-infected mice and is the cause of the high mortality rate.^24^ However, the H5N8 virus distribution in infected mice was limited in brain, lung, heart, spleen tissues.^20^ Therefore, we investigated whether the altered virulence of the single gene reassortant corresponded with the ability to replicate in additional organs including brain, turbinate, kidney, spleen, heart, liver, and intestine. The H5N8 (W452) virus was detected in lungs (1, 3, 5 and 7 dpi (5/5)), brains (5 dpi (4/5) and 7 dpi (3/5)), nasal turbinate (1, 3, 5 dpi (5/5), 7 dpi(2/5)) hearts (3 dpi (5/5)), and spleen (5 dpi (4/5) and 7 dpi(3/5)) while the H5N1 (W149) virus was detected to high levels in all tissues tested from all animals (Fig. 2 and Supp. Fig. 1). Most of the single gene reassortant viruses categorized into moderately and low-pathogenic groups were sporadically detected in multiple organs in addition to upper (nasal turbinate) and lower (lung) respiratory tissue; however, the virus titer was lower than that of the H5N1 (W149) virus (Fig. 2 and Supp. Fig. 1). All viruses exhibited higher titers in the lung than in the nasal turbinate (Fig. 2A to C vs Fig. 2D to F) suggesting the reassortant virus might have relatively high tropism for the lower respiratory tract. Interestingly, however, the W452_W149PB2_ virus was detected in all organs tested. These results clearly demonstrate that the PB2 gene from the HPAI H5N1 (W149) virus substantially alters virus replication and expand the tissue tropism in the infected host.

### Correlation between differential cytokine expression in BALF of mouse lungs and virus pathogenicity

**Figure 3.**
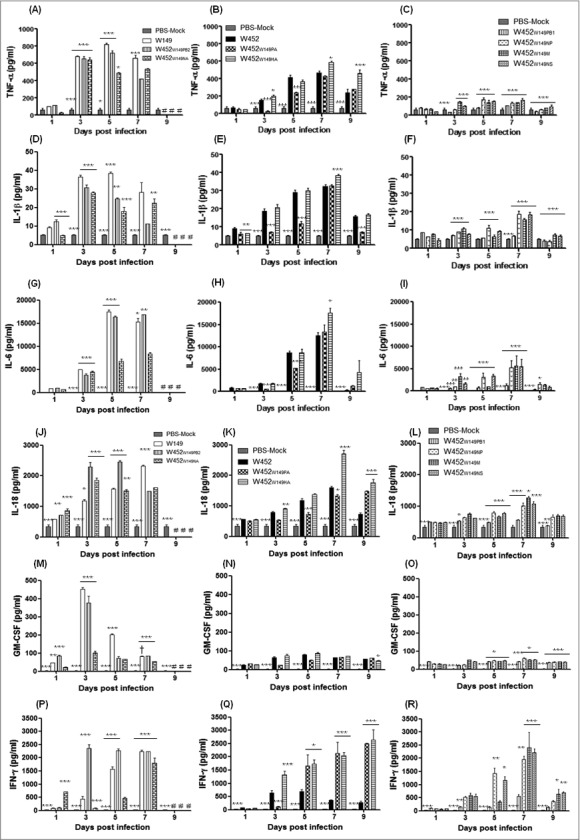
(see previous page) Cytokine and chemokine responses in infected lungs. Concentrations of various cytokines/chemokines were measured in BAL fluids of infected mice at 1, 3, 5, 7, and 9 dpi by microbead suspension assay with the Luminex-based multiplex immunoassay kit (Affymetrix, Santa Clara, CA, USA). (A-C) TNF-α, (D-F) IL-1β, (G-I) IL-6, (J-L) IL-18, (M-O) GM-CSF, and (P-R) IFN-γ are indicated. (A), (D), (G), (J), (M), and (P) were collected from highly pathogenic viruses. (B), (E), (H), (K) (N) and (Q) were from moderately pathogenic viruses. (C), (F), (I), (L), (O) and (R) were from low-pathogenic viruses. The values shown are means ± SD (errors bars) from 5 mouse BAL fluid samples per time point tested. #, No samples collected because the mice in this group died. Asterisks indicate the statistical significance between W452 and other viruses determined by One-way ANOVA. Further, daggers indicate the statistical significance across time points. (* or † indicates *p* < 0.05, ** indicates ** p < 0.01, and *** indicates *p* < 0.001).

To elucidate the relationship between cytokine production and viral pathogenicity, we measured induction of cytokines in the bronchoalveolar lavage (BAL) fluid of infected mice at 1, 3, 5, 7, and 9 dpi (Fig. 3 and Supp. Fig. 2). Although some significant differences were evident at 1 dpi in the highly pathogenic group, the expression levels of most cytokines showed significant differences by 3 dpi, peaked at, peaked at 5 dpi, and were then maintained or gradually decreased until 7 dpi, depending on the virus. Proinflammatory cytokines (tumor necrosis factor (TNF)-α, interleukin (lL)-1β, IL-6, IL-18, and GM-CSF) exhibited higher expression at early time points (3 to 5 dpi) in the highly pathogenic group (W149, W452_W149PB2_, and W452_W149NA_) compared with the other 2 groups (Fig. 3A, D, G, J, and M). The viruses in the moderately pathogenic group (W452, W452_W149PA_ and W452_W149HA_) induced low to intermediate expression of cytokines with relatively delayed kinetics (5 to 7 dpi) with the expression levels gradually increasing up to 7 dpi (Fig. 3B, E H, K, and N). However, the viruses categorized as low pathogenic (W452_W149PB1,_ W452_W149NP_, W452_W149M,_ and W452_W149NS_) showed low levels of cytokine expression compared with W452 at 5 dpi (Fig. 3C, F, I, L, and O). Interestingly, expression of IFN**-**γ, a proinflammatory cytokine, was elevated in all groups of infected mice, especially in those infected with the highly pathogenic viruses where it was found to be elevated from 7 dpi up until all the mice succumbed to death (Fig. 3P–R). IFN-γ was also upregulated in mice infected with moderately and low pathogenic viruses, with the exception of the wild-type W452 virus, which induced only low expression levels of this cytokine compared with all other viruses tested (Fig. 3Q). While IL-12p70 largely exhibited similar expression patterns with the above mentioned cytokines, W452_W149PB2_ and W452_W149NA_-infected mice showed markedly higher levels of IL-12p70 (even compared with parental W149 virus) from 3 dpi until death (Supp. Fig. 2A-C). Moreover, the expression of IL-15 and MIP1β were highest at 3 dpi in the highly pathogenic viruses while the moderately and low-pathogenic virus-infected mice showed mildly elevated to normal expression levels (Supp. Fig. 2D-I). Further, expression of IL-2, IL-5, and IL-13 showed increases during the virus infections in each group (Supp. Fig. 2J-R). Together these data suggest that elevated expression of pro-inflammatory cytokines and chemokines is closely associated with the high virulence of the W452_W149PB2_ and W452_W149NA_ reassortants.

### Increased histopathological damage to mouse lungs caused by reassortant viruses

**Figure 4.**
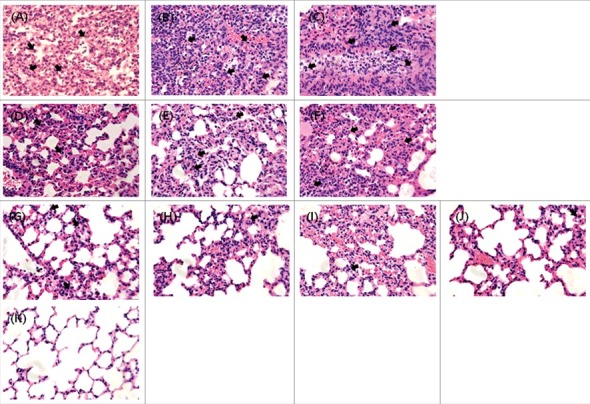
Histopathology of lungs from mice infected with each reassortant virus. Mice were inoculated intranasally with 105.5 EID50 of each virus. Lungs were harvested on day 6 after inoculation. Regions of inflammatory lesions were indicated by arrows. (A) W149 (A/EM/Korea/W149/2006), (B) W452W149PB2, (C)W452W149NA, (D) W452 (A/MD/Korea/W452/2014), (E) W452W149PA, (F) W452W149HA, (G) W452W149PB1, (H)W452W149NP, (I) W452W149M, (J)W452W149NS, and (K) mock-infected. Magnification x400.

To correlate virulence with pathogenicity, lung tissues from inoculated mice were collected at 6 dpi for pathological assessment. Viruses categorized into increased pathogenic groups (W149, W452_W149PB2_, and W452_W149NA_) showed histopathological damage, including serious exudate-filled alveolar spaces (Fig. 4A–C). Of note, W452_W149NA_ demonstrated the most extensive infiltration of inflammatory cells (Fig. 4C). Further, immunostaining with NP antigens clearly showed the virus-infected cells (Supp. Fig. 3). Viruses categorized into the moderately pathogenic group (W452, W452_W149PA_ and W452_W149HA_) demonstrated inflammation in limited areas of the lungs (Fig. 4D–F). In contrast, lungs infected with low-pathogenic viruses showed only small, contained areas of inflammatory infiltrates and minimal increases in cellularity within the interstitial tissue (Fig. 4G–J).

### Enhanced polymerase activity in increased pathogenic viruses

**Figure 5.**
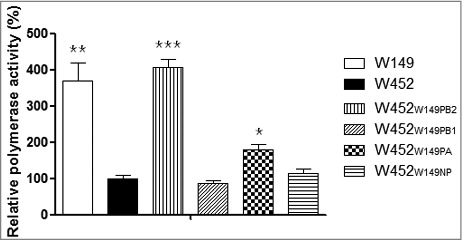
Enhanced polymerase activity in reassortant RNPs. The polymerase activities of reconstituted RNP complexes composed of the PB2, PB1, PA, and NP plasmids from W452 and W149 strains. The polymerase activity test values are the mean values of at least 3 independent assays. Asterisks indicate samples significantly different from the W452 RNP complex as determined by one-way ANOVA and subsequent Dunnett's test. Further, daggers indicate the statistical significance across time points. (* indicates *p* < 0.05, ** indicates ** p < 0.01, and *** indicates *p* < 0.001).

The polymerase activity of the influenza virus vRNP complex, PB2, PB1, PA and NP, is engaged in replication and transcription.^25^ To determine the correspondence of this polymerase activity to the results of the virulence study in mice, we exploited a mini-genome reporter assay which detects any potential contribution of the vRNP complex genes individually (Fig. 5). Various combinations of W149 PB2, PB1, PA, or NP were examined in the backbone of W452. The RNP complexes with the PB2 or PA of W452 replaced with that of W149 resulted in increased polymerase activity (4 and 1.7-fold, respectively). However, replacement of the W452 genes with PB1 and NP from W149 could not significantly reduce or elevate polymerase activity (0.8 and 1.1-fold, respectively). Taken together, these results demonstrate that replacing W452PB2 with W149PB2 in the vRNP complex is sufficient to dramatically increase the polymerase activity in the mammalian 293T cells, which becomes equal to that of the W149 polymerase.

### In vitro growth properties of reassortant viruses in mammalian cells

**Figure 6.**
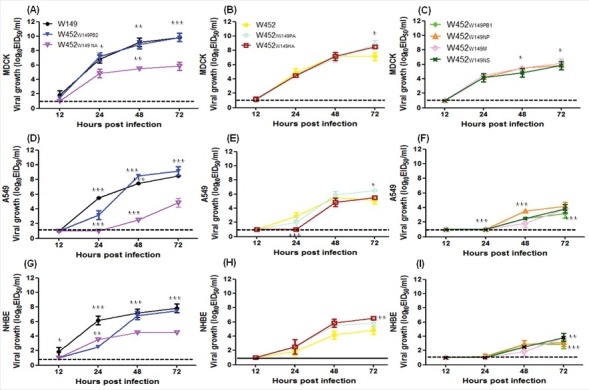
Comparative growth curves in MDCK, A549, and NHBE cells after infection with parental and reassortant viruses. (A-C) MDCK, (D-F) A549, and (G-I) NHBE cells were infected with 0.01 or 0.1 MOI and incubated at 35°C. Supernatants were collected at 12, 24, 48, and 72 hpi for viral titer determination. Standard deviations are indicated from 3 independent experiments. Asterisks indicate the statistical significance between W452 and other viruses determined by one-way ANOVA and subsequent Dunnett's test. (* indicates *p* < 0.05, ** indicates ** p < 0.01, and *** indicates *p* < 0.001).

To examine the *in vitro* growth properties of each gene reassortant, we analyzed their multistep growth curves in MDCK, A549 (adenocarcinomic human alveolar basal epithelial cells), and normal human bronchial epithelial (NHBE) cells (Fig 6). Viral titers with W149 and W452_W149PB2_ viruses showed at least 100-fold higher mean peak titers (*p* < 0.05) than W452 in all infected cells. In MDCK cells, W452_W149PB2_, and to a lesser extent the viruses possessing the PA or HA of W149 replicated more efficiently than parental W452 virus (peak titers at 72h p.i. of 10^8.5^ to 10^9.75^ Vs 10^7.25^ EID_50_/ml, whereas the W452_W149PB1_, W452_W149NP_, W452_W149NA_, W452_W149M_, and W452_W149NS_ viruses demonstrated attenuated replication kinetics (10^5.75^ to 10^6.25^ EID_50_/ml mean peak titer) (Fig 6A-C).

In 2 systems of human cells (lung epithelial A549 and NHBE), these viruses demonstrated delayed and reduced titers compared with those of MDCK cells (Fig 6D to I). In A549 cells, the W149, W452_W149PB2_, and W452_W149PA_viruses reached higher mean peak titers (10^6.5^ to 10^9.25^ EID_50_/ml) than the W452 virus (10^5.25^ EID_50_/ml) at 72 hours post infection (hpi) (Fig 6D-E). Moreover, W452_W149HA_ and W452_W149NA_ demonstrated similar peak titers to W452 (*P*>0.05), and the other viruses showed reduced mean peak titers (10^2.75^ to ^4.25^ EID_50_/ml) (Fig 6F). Viral titers in the NHBE cells showed similar patterns with those in A549 cells(Fig 6 G-I). Interestingly, even though the viral mean peak titer (at 72h p.i.) was similar to that of the W452 virus, W452_W149NA_ demonstrated higher titers (10^3.5^ EID_50_/ml) at 24 h p.i. Overall, these results demonstrate that in the W452 backbone a single gene substitution from W149 can alter the *in vitro* growth characteristics of H5N8 (W452) virus in the 3 mammalian cell lines examined.

### Replication properties of increased pathogenic reassortant viruses in ferrets

In mice the W452_W149PB2_, W452_W149HA_, and W452_W149NA_ viruses demonstrated increased virulence with low MLD_50_ compared with the other reassortant viruses. Since the ferret is recognized as the most suitable host for influenza virus studies, the W452_W149PB2_, W452_W149HA_ and W452_W149NA_ viruses were intranasally inoculated into groups (n = 15) of ferrets at 10^6^ EID_50_ to investigate whether these viruses could efficiently replicate and be pathogenic in this model. One day after virus infection, 3 naïve ferrets were added into cages adjacent to infected animals to evaluate any possible indirect droplet transmission. For comparison, we also tested W149 and W452 viruses. Nasal washes from each group of ferrets were collected at 1, 3, 5, and 7 dpi for virus titration. In addition, 3 ferrets from each group were killed on 1, 3, 5, and 7 dpi (3 ferrets/dpi) to assess the viral titers and tissue distribution of the virus.

**Figure 7.**
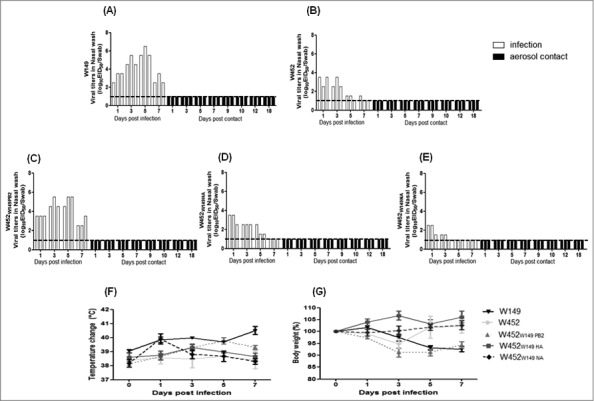
Viral shedding, weight loss, and body temperature in ferrets inoculated with reassortant viruses. Groups of ferrets (n = 3/group) were inoculated intranasally with 10^6^ EID_50_ of virus. To examine transmission, the inoculated animals were individually paired with an aerosol-contact animal (1:1 setup, in triplicate) at 1 dpi and monitored for virus shedding in nasal swabs. (A) W149 (B) W452, (C) W452_W149PB2_, (D) W452_W149HA_, and (E) W452_W149NA_. The limit of detection was 1 log_10_ EID_50_/Swab and is indicated by the dotted line for each representation. After the inoculation of virus, temperature (F) and body weight loss (G) were measured every other day. Temperature is represented as a °C and weight change is demonstrated as a percentage of the initial body weight.

Wild-type W149 and W452 showed clear differences in growth kinetics in various organs including nasal washes. The W149 virus was detected in nasal washes of infected ferrets until 7 dpi and reached a mean peak titer of 10^5.8^ EID_50_/Swab at 5 dpi, while the W452 virus showed the highest virus mean peak titer at 1 dpi (10^3.1^ EID_50_/Swab), which gradually decreased until 7 dpi (only one ferret showed a titer of 10^l.5^ EID_50_/Swab(1/3)) (Fig. 7A and B). Furthermore, W149-infected ferrets showed increased body temperature (> 1.0°C increase in body temperature) with 5–8% of body weight loss, while W452 infected ferrets showed no detectable clinical signs (< 0.9°C increase in body temperature and less than 2% of body weight loss) (Fig. 7F and G). Interestingly, the W452_W149PB2_ demonstrated the highest mean nasal wash peak titers (10^5.1^ EID_50_/Swab at 5 dpi) with levels at 7 dpi of 10^2.8^ EID_50_/Swab (Fig 7C). Further, these ferrets exhibited comparable increases in body temperature and weight loss to W149 (H5N1)-infected ferrets (Fig. 7F and G). However, W452_W149HA_ and W452_W149NA_ viruses showed similar or decreased nasal wash titers compared with W452 virus (Fig. 7D and E). Further, although there was an increase in body temperature in ferrets infected with W452_W149NA_ virus at 1 dpi, the animals rapidly recovered (Fig 7F and G)

.

None of the nasal washes from contact ferrets in any group were positive for virus during the duration of experiment (18 days) (Fig. 7A–E). Moreover, serologic testing (Hemagglutination Inhibition (HI) assay) of contact ferret sera showed no detectable HI titers against virus inoculums at 18 dpc (data not shown).

**Figure 8.**
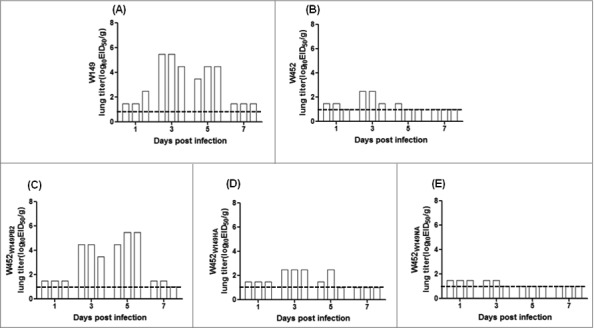
Lung viral titers in ferrets inoculated with (A) W149, (B) W452, (C) W452_W149PB2_, (D) W452_W149HA_, and (E) W452_W149NA_. Groups of ferrets were inoculated intranasally with 10^6^ EID_50_ of virus and killed at 1, 3, 5, 7 dpi (n = 3/group). The limit of detection was 1 log_10_ EID_50_/Swab and is indicated by the dotted line for each representation.

In a previous study, we showed that the W452 virus possesses only low replication properties in tissues outside the respiratory tract of infected ferrets.^20^ Hence, we examined whether altered virulence of W452_W149PB2_, W452_W149HA_, and W452_W149NA_ viruses could also influence virus replication properties outside of the respiratory organs. In agreement with the previous study, the H5N1(W149) virus was recovered from organs outside the respiratory tract, such as spleen, kidney and intestine (Fig 8A and Table 2), while W452 (H5N8) virus showed relatively restricted replication limited to brain, kidney, and intestine compared with H5N1(W149) (Fig 8B and Table 2). Interestingly, the W452_W149PB2_ virus showed increased viral titers compared with the W452 virus at 3, 5, and 7 dpi in brain, turbinate, trachea, lung, heart, and intestine (Fig 8C and Table 2). Of note, the W452_W149PB2_ virus showed comparable lung virus titers and 10 times higher tracheal titers at 3 dpi compared with those of W149 virus. Further, W452_W149PB2_ viruses were recovered from the hearts and brains of infected ferrets at titers as high as 10^2.2^ to 10^2.8^ EID_50_/g. However, W452_W149HA_ and W452_W149NA_ viruses showed only restricted viral titers in the respiratory tract (turbinate, trachea, and lung) with comparable or decreased titers compared with W452 virus (Fig 8D–E and Table 2). These results demonstrate that the W452_W149PB2_ virus could increase the viral tropism as well as growth properties in infected mammalian hosts.

**Table 2.** Viral titers in various organs and tissues in ferrets.

	^a^W149				W452				W452_W149PB2_				W452_W149HA_				W452_W149NA_			
	1dpi	3dpi	5dpi	7dpi	1dpi	3dpi	5dpi	7dpi	1dpi	3dpi	5dpi	7dpi	1dpi	3dpi	5dpi	7dpi	1dpi	3dpi	5dpi	7dpi
brain	—	—	—	—	—	2.1 ± 0.6	1.2 ± 0.3	—	—	2.8 ± 0.6	2.2 ± 0.6	—	—	—	—	—	—	—	—	—
Turbinate	^b^2.1 ± 0.6	2.8 ± 0.6	1.5	1	2.2 ± 0.6	2.8 ± 0.6	—	—	3.2 ± 0.6	3.8 ± 0.6	3.5	1.8 ± 0.6	2.1 ± 0.6	2.8 ± 0.6	1.3 ± 0.3	—	1.5	1.8 ± 0.6	—	—
trachea	1.3 ± 0.3	1.8 ± 0.6	2.8 ± 0.6	—	1.3 ± 0.3	1.8 ± 0.6	—	—	1.5 ± 0.6	2.8 ± 0.6	2.2 ± 0.5	1.3 ± 0.3	1.5	—	—	—	1.5	—	—	—
heart	—	—	—	—	—	—	—	—		2.2 ± 0.6	—	—	—	—	—	—	—	—	—	—
spleen	—	2.1 ± 0.6	1.3 ± 0.3	—	—	—	—	—	—	—	—	—	—	—	—	—	—	—	—	—
kidney	—	2.8 ± 0.6	1.8 ± 0.6	1.2 ± 0.3	—	1.3 ± 0.3		—	—	—	—	—	—	—	—	—	—	—	—	—
intestine	—	1.2 ± 0.3	—	—	—	1.3 ± 0.3	1.2 ± 0.3	—	—	1.8 ± 0.6	1.3 ± 0.3	—	—	—	—	—	—	—	—	—

*Notes.* Dashed lines indicate that the tissue was negative for virus detection (lower limit = 1 log_10_EID_50_/g).

a Results were obtained from 3 animals per group at each time point and are expressed as log_10_ EID_50_/g tissue.

b Standard deviation titers.

### Increased pathogenicity of reassortant viruses in ferret lungs

**Figure 9.**
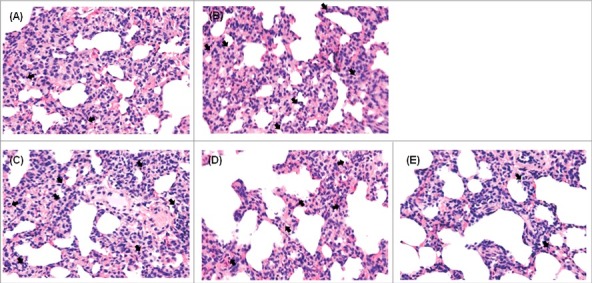
Histopathology of lungs from ferrets infected with highly pathogenic viruses. Ferrets were inoculated intranasally with of 106 EID50 of each virus. Lungs were harvested on day 5 after virus inoculation followed by sectioning and H&E staining. Regions of inflammatory lesions were indicated by arrows. (A) W149, (B) W452, (C) W452W149PB2. (D) W452W149HA (E) W452W149NA. W149 or W452W149PB2 inoculated ferrets showed more inflammation than W452. Magnification x400.

To correlate virulence with pathogenicity, lung tissues from inoculated ferrets were collected at 5 dpi (Fig. 9). Lungs inoculated with W149, W452_W149PB2_ showed more visible macroscopic lesions (Fig. 9A, and C). However, the lungs of the ferrets infected with W452 displayed only moderate inflammation compared with those infected with W149 and W452_W149PB2._ Taken together, these results demonstrate that infection with W452_W149PB2_ results in increased pathogenicity in the lungs of ferrets.

### Discussion

An outbreak of novel reassortant HPAI H5N8 virus, which is a member of clade 2.3.4.4, was reported in 2014 in South Korea and subsequently spread into nations in Asia (China and Japan), Europe (Russia, Italy, United Kingdom, Netherlands, Germany), and North America (Canada and the United States).^10,12-14^ Generally, the H5N1 subtype was stably maintained for more than a decade before it started to evolve into the novel reassortant HPAI H5Nx virus in 2008.^26^ However the clade 2.3.4 H5 HPAIVs have been reported with different NA subtypes (H5N1, H5N5, and H5N8) in China,^4,13,27^ and have been continuously found in the USA (H5N1, H5N2, and H5N8) implying that this virus is still actively evolving by switching genes with other avian viruses. Thus, although the H5N8 virus exhibited only low to moderate virulence in mammalian hosts compared with H5N1^20^, we investigated whether active reassortment could alter its virulence. To this end, we demonstrate that HPAI H5N8 could easily integrate genes from circulating HPAI H5N1 (clade 2.2, W149) viruses without any genetic incompatibility since all 8 combinations of single-gene reassortants were selected from virus co-infections of MDCK cells by plaque purification

Further, some reassortant viruses showed enhanced growth properties *in vitro*, increased virulence, an expanded range of tissue tropism, and *in vivo* growth properties at levels similar to those of H5N1(W149). This was especially true of the H5N8 (W452) virus containing the PB2 gene from W149 virus. In fact, the W452_W149PB2_ virus showed 1,000-fold higher virulence than W452 virus (MLD_50_ = 10^2.5^ vs 10^5.8^ EID_50_, respectively) and 10–100 fold higher viral lung titers at all-time points in mice with systemic infection. Reciprocally, the W149_W452PB2_ virus showed more than 10-fold attenuation in virulence compared with W149 virus (MLD_50_ = 10^4.5^ vs 10^3.3^ EID_50_, respectively) (Table 1). Many studies reported that the E627K mutation in PB2 facilitates the adaptation of avian viruses to mammals and increases pathogenesis.^28-31^ In our study, substitution of the W149 PB2 containing 627K dramatically increased viral pathogenesis in mice and ferrets. Therefore, our data are in agreement with the increased virulence for mammals conferred by the E627K substitution. However, we cannot rule out the possible roles of 147T and 339T in W149 PB2, which also might contribute to the increased lung titers in mice.^32^ In addition, full amino acid sequence comparison revealed 96.44% homology (27/759) between the 2 strains (Supp Fig 4).^28,29,31,32^ Therefore, more detailed molecular studies are crucial to understand the virulence mechanism of W452_W149PB2_ virus using reverse genetic methods because the virulence of influenza viruses are affected by polygenic factors.^33^ Nevertheless, our results clearly demonstrate that a single gene substitution (PB2 in this study) could alter the virulence of recent wild spread H5N8 viruses.

In China, an H5N1 inactivated vaccine has been compulsorily used to control widespread HPAI H5N1 throughout the country.^34^ Presumably because the recent H5N1 vaccine (Re-5) is representative of clade 2.3.4, there has been increased detection of diverse sublineages, including clade 2.3.4.4–6, which showed low cross reactivity with the recent vaccine strain.^35^ Interestingly, the H5 clade 2.3.4.4 viruses possess various subtypes of NA genes other than the N1 subtype.^6,11,36^ Under the selective pressure of a vaccine, viruses could alter their receptor binding affinity through their HA genes and this may cause reduced genetic compatibility between H5 and N1 subtypes, which has been generally shown to be a stable subtype. The HA and NA proteins both engage a glycan receptor on host cells and their interplay has important inference for viral fitness and proper replication by the functional balance of HA binding and NA cleavage.^37^ One of the reassortant viruses in the current study, W452_W149NA_, showed relatively low viral titers in eggs (10–100 fold lower than the other reassortant) and viral growth, but was categorized into the highly pathogenic group due to the low MLD_50_ (4.8 log_10_EID_50_). Further study will be necessary to verify this, but it appears likely that HPAI H5 viruses belonging to clade 2.3.4.4 may begin to switch their neuraminidase gene with that of other viruses to obtain one more suitable for their HA and NA balance, such as H5N8.^38^

The unbalanced production of inflammatory cytokines and chemokines observed during influenza virus infections has been suggested to be a mechanism of pathogenesis for these viruses.^39^ Several studies demonstrated that early rise of TNF- α, IL-1 and IL-6 and the resulting cascade of chemokines in BAL fluid or lung homogenates results in the accumulation of neutrophils in the lung and formation of lung pathology.^40-42^ This mechanism has been termed a *cytokine storm* and is followed by massive innate immune responses associated with acute respiratory distress syndrome, a typical medical condition of highly pathogenic influenza infection that results in high morbidity and mortality.^43,44^ In this study, 3 patterns of cytokine secretion were observed depending on the level of pathogenicity. For example, extensive secretion of almost all inflammatory cytokines tested was shown in the highly pathogenic group at early time points, while low levels and delayed cytokine secretions were observed in the low pathogenic group at most time points, implying that aberrant cytokine secretion, especially at early time points, induces high mortality. Moreover, incremental secretion of cytokines over time was observed in the moderate pathogenic group and correlated with death at later time points than that seen with the highly pathogenic viruses. Although viral lung titers and systemic infection in mice generally correlated with virulence, the patterns of cytokine secretion caused by viral infection may also be another biologic indicator of pathogenesis.

To verify the increased virulence and growth properties of the W452_W149PB2_ virus in mammalian hosts, we performed ferret infection and indirect transmission studies comparing these to wild-type W149 and W452 viruses. Similar to the results of the mouse study, the W452_W149PB2_ virus exhibited increased virus titers in nasal washes compared with the W452 virus; however, there was no evidence of aerosol transmission to naive contact ferrets (Fig. 7). Further, the W452_W149PB2_ virus induced significantly high and prolonged virus titers in infected ferrets as well as wide organ distribution compared with the W452 virus (Fig. 8 and Table 2). Interestingly, although the W149 virus (HA clade 2.2) was not detected in brain tissue, the W452_W149PB2_ and W452 viruses (HA clade 2.3.4.4) were (10^1.2^ to 10^2.8^ EID_50_/g) (Table 2). These results suggest that W452_W149PB2_ virus has similar characteristics as the W452 virus including enhanced growth properties and pathogenic potential in ferrets. Further, W452_W149PB2_ virus also exhibited enhanced virus titers in 2 systems of human cells (lung epithelial A549 and NHBE) suggesting that such a reassortant is a potential threat to public health (Fig. 6).

The current study focused on the investigation of virulence properties of the reassortant H5N8 viruses in mice and ferrets, which are appropriate models for the evaluation of viral pathogenesis.^45^ Previously, we reported that the H5N8 virus induces only mild to asymptomatic clinical signs in domestic ducks suggesting that this host may play a critical role as a *Trojan horse* and mixing vessel for the creation of novel reassortants with pandemic potential. Furthermore, the rapid and wide spread of H5N8 virus to Asia and Europe and the first outbreak of these viruses in North America has the potential for the creation of novel viruses with newly-introduced genes.^46^ Our study demonstrates the pathogenic potential of H5N8 reassortant viruses with newly introduced genes from other avian influenza viruses and emphasizes the need for intensive monitoring of reassortment events with co-circulating avian or mammalian viruses to design vaccines as part of pandemic preparedness.

## Materials and methods

### Reassortant viruses

To obtain reassortant viruses containing single gene changes from A/EM/Korea/W149/06[W149(H5N1)] into the A/MD/Korea/W452/2014[W452(H5N8)] backbone, the 2 viruses were mixed (1:3 ratio) and was used to infect MDCK (Madin-Darby canine kidney, ATCC) cells, and then plaque purification was conducted. Briefly, a monolayer of MDCK cells in a 6-well plate were infected with serially diluted viruses for 1 hour, washed twice with phosphate buffered saline (PBS, Lonza, Swiss) and overlaid with a 0.7% agarose-EMEM media with or without L-1-tosylamide-2-phenylmethyl chloromethyl ketone (TPCK)-treated trypsin (Worthington Biochemical Corporation, NJ, USA). After 3 d of incubation, plaque colonies were picked and resuspended in medium and injected into 10-day-old embryonated chicken eggs (Choong Ang Vaccine Laboratories Corporation, Daejeon, Korea). After 30 hours of incubation, viruses were harvested and full-length gene segments sequenced to confirm the origin of each segment. Confirmed viruses were stored at -80°C and titrated via the EID_50_ end point. The handling of viruses was performed in an enhanced biosafety level 3 (BSL3) containment laboratory as approved by the Korean Centers for Disease Control and Prevention (KCDC-14–3–07).

### Experimental infection in mouse

To determine the 50% mouse lethal dose (MLD_50_) of each virus, 8-week-old female BALB/c mice (Samtaco, n = 10 per group) for each virus were anaesthetized and inoculated intranasally with 10-fold serially diluted virus in a 30 ul volume. The mice were monitored daily for 14 d and checked for changes in body weight. The infected mice were killed if they lost more than 25% of their initial body weight.

Additionally, groups of mice (n = 38 per group) were inoculated intranasally with 10^5.5^EID_50_ of each virus. Ten mice in each group were monitored daily for up to 14 d for weight change, morbidity, and mortality. Five mice from each inoculated group were killed on 1, 3, 5, 7, and 9 dpi for collection of BAL fluids and virus detection in various tissues (lung, heart, liver, spleen, kidney, intestine, and brain). Tissues were homogenized with an equal volume (ml/g) of PBS containing antibiotics (1% penicillin and streptomycin, Gibco). Homogenates were cleared by centrifugation, and then viral titers in the 10-fold serial dilutions of supernatants were measured by determining the EID_50_. The remaining mice were used for H&E staining at 6 dpi.

### Expression of cytokines and chemokines in BAL fluid

BAL fluid from mouse lungs was collected at 1, 3, 5, 7, and 9 d after each virus infection or from the uninfected control group. The BAL solution was then centrifuged at 12,000 rpm for 5 min at 4°C and stored at −80°C until the analysis.

A multiplex biometric immunoassay was adopted for cytokine measurement according to the manufacturer's instructions (Procarta cytokine assay kit; Affymetrix, Santa Clara, California, USA), The concentrations of TNF-α, lL-1β, IL-6, IL-12p70, IL-15/IL15R, INF-γ, IL-18, IL-2, IL-5, IL-13, GM-CSF, and MIP-1β were measured. Briefly, BAL fluid samples were incubated with antibody-coupled beads corresponding to the various cytokines. Complexes were washed, then incubated with biotin-conjugated secondary antibody, followed by another wash and incubation with phycoerythrin reporter conjugated-streptavidin. Assays were measured by detection of the fluorescent reporter signal and identification of beads on a Bio-Plex 200 system and data were analyzed using the associated Bio-Plex Manager software version 4.1.1 (Bio-Rad Laboratories, Hercules, CA, USA).

### Polymerase activity assay

Luciferase mini-genome reporter assays were performed using a luciferase reporter plasmid (pHW72-Luci) containing the influenza A virus M – driven noncoding region.^23^ Briefly, 293T cells were prepared and were transfected with pHW2000-PB2, pHW2000-PB1, pHW2000-PA, pHW2000-NP, pHW72-Luci, and pCMV-β-galactosidase plasmids using TransIT-LT1 transfection reagent. After 8 h the media was changed to DMEM (Gibco) containing 7% FBS. After 24 h the cells were washed with PBS and lysed using reporter lysis buffer for 20 mins (Promega). Cell lysates then were harvested in 96-well plates and luciferase activities were measured using the luciferase assay system (Promega). The results were normalized to the β –galactosidase activity of the cells.

### Cell and virus replication

MDCK, A549 (human lung adenocarcinoma epithelial cell line, KCLB, Korea) and NHBE cells were cultured in 6-well plates and then infected with reassortant viruses at a multiplicity of infection (MOI) of 0.01 or 0.1 for 1 hour, washed and incubated at 35°C in a 5% CO2 atmosphere in the presence of TPCK-treated trypsin (2 ug/ml). Cell culture supernatants were collected at 12, 24, 48, and 72 hpi and virus titers were determined by EID_50_.

### Experimental infection of ferrets

Female ferrets, 18- to 20- weeks old and sero-negative for influenza A viruses (ID Bio Corporation) (n = 15/group), were intranasally inoculated with 10^6.0^ EID_50_ of W452, W149 and W452_W149PB2_ viruses under anesthesia. Three ferrets per group were killed at 1, 3, 5, and 7 dpi for virological and pathogenesis experiments. After 24 h, naïve ferrets (n = 3) were co-housed in the other half of a cage (individual cage) with a ferret in each inoculated group, with the ferrets only separated by 2 stainless steel grids allowing for airborne virus transmission. Ferret body weight and temperature were monitored every other day until 7 dpi. Nasal washes were collected every other day from the inoculated ferrets and daily from 1 day post-exposure from the contact ferrets until 18 d post contact (dpc) and blood was collected from contact groups at 18 dpc. Virus titrations of nasal washes and various tissue organs were determined by EID_50_ assays.

### Histopathology

Mouse lungs infected with each virus and ferret lungs infected with W452, W149, W452_W149PB2_ W452_W149HA_, and W452_W149NA_ viruses were harvested at 6 dpi or 5 dpi, respectively. The samples were fixed in 10% neutral-buffered formalin and embedded in paraffin. Histological assessment was conducted using standard hematoxylin and eosin staining and light microscopy (magnification x400) or stained with NP polyclonal antibodies. The slides were viewed using an Olympus η 71 (Olympus, Tokyo, Japan) microscope and DP controller software to capture images.

### Statistical analysis

To assess significant differences in viral titers and cytokine expression levels between groups or across time points W452-infected samples were compared. Asterisks indicate the statistical significance between W452 and other viruses determined by One-way ANOVA and subsequent Dunnett's test. Further, daggers indicate the statistical significance across time points. * or † indicates *p* < 0.05, **or †† indicates ** *p* < 0.01, and *** or ††† indicates *p* < 0.001). All statistical analyses were performed with using GraphPad Prism version 5.00 for Windows (GraphPad Software, La Jolla, CA).

### Ethics statement

All animal experiments, including use of embryonated chicken eggs, were approved by the Medical Research Institute, a member of Laboratory Animal Research Center of Chungbuk National University (LARC) (approval number: CBNUA-954–16–01) and were conducted in strict accordance and adherence to relevant policies regarding animal handling as mandated under the Guidelines for Animal Use and Care of the Korea Center for Disease Control (K-CDC).^48^ Specific pathogen-free 10-day-old embryonated chicken eggs were purchased from Choong Ang Vaccine Laboratories Corporation, Daejeon in South Korea. The generation of recombinant viruses (LMO-CBNU-7108) and handling of viruses were performed in an enhanced biosafety level 3 (BSL3) containment laboratory as approved by the Korean Centers for Disease Control and Prevention (KCDC-14–3–07).

## Supplementary Material

KVIR_S_1366408.zip
