# Supplementary material for: Altered virulence of Highly Pathogenic Avian Influenza (HPAI) H5N8 reassortant viruses in mammalian models
Source: Virulence. 2017 Sep 21;9(1):133–48. doi: 10.1080/21505594.2017.1366408 (PMC5955454; doi:10.1080/21505594.2017.1366408)
Supplement: KVIR_S_1366408.zip [file kvir-09-01-1366408-s001.zip › KVIR_S_1366408.docx]

**Supporting Information**

**Supplementary Figure Legends**

**Figure S1.** Replication of reassortant viruses in mouse tissues. After infection with each virus, samples were collected on 1, 3, 5, 7, and 9 dpi. Viral titers from (A – C) heart, (D – F) liver, (G – I) spleen, (J – L) kidney, and (M – O) intestine. (A), (D), (G), (J), and (M) were collected from mice infected with highly pathogenic viruses. (B), (E), (H), (K), and (N) are from mice infected with moderately pathogenic viruses. (C), (F), (I), (L), and (O) are from mice infected with low-pathogenic viruses. Viral titers shown are means ± SD from five animals per time point and titers below the limit of detection are shown as 1 log_10_EID_50_/g. The results were confirmed by two independent experiments #, No samples collected because the mice in this group died. Asterisks indicate the statistical significance between W452 and other viruses determined by one-way ANOVA. Further, daggers indicate the statistical significance across time points. (* or † indicates *p* < 0.05, **** indicates *** p* < 0.01, and *** or ††† indicates *p* < 0.001).

**Figure S2.** Cytokine and chemokine responses in infected lungs. Concentrations of various cytokines were measured in BAL fluids of infected mice at 1, 3, 5, 7, and 9 dpi. (A – C) IL-12p70, (D – F) IL-15/IL-15R, (G – I) MIP1β, (J – L) IL-2, (M – O) IL-5, and (P - R) IL-13. (A), (D), (G), (J), (M), and (P) are collected from mice infected with highly pathogenic viruses. (B), (E), (H), (K), (N), and (Q) are from mice infected with moderately pathogenic viruses. (C), (F), (I), (L), (O), and (R) are from mice infected with low-pathogenic viruses. The values shown are means ± SD (errors bars) from BAL fluid samples from five mice per time point. #, No samples collected because the mice in this group died. Asterisks indicate the statistical significance between W452 and other viruses determined by one-way ANOVA. Further, daggers indicate the statistical significance across time points. (* or † indicates *p* < 0.05, ***or* indicates *** p* < 0.01, and *** indicates *p* < 0.001).

**Figure S3.** IHC of lungs from mice infected with each reassortant virus. Mice were inoculated intranasally with 10^5.5^ EID_50_ of each virus. Lungs were harvested on day 6 after inoculation. (A) W149 (A/EM/Korea/W149/2006), (B) W452_W149PB2_, (C)W452_W149NA_, (D) W452 (A/MD/Korea/W452/2014), (E) W452_W149PA_, (F) W452_W149HA_, (G) W452_W149PB1_, (H)W452_W149NP_, (I) W452_W149M_, (J)W452_W149NS_, and (K) mock-infected. Presence of influenza NP antigen was determined by IHC and marked with arrows. Magnification x400.

**Figure S4.** Comparison of the amino acid sequences of the influenza virus PB2 gene. Alignment analysis. Amino acids were analyzed using the DNAStar Lasergene sequence analysis software package, version 5.0 (DNAStar, Madison, WI).
