# Supplementary material for: Altered virulence of Highly Pathogenic Avian Influenza (HPAI) H5N8 reassortant viruses in mammalian models
Source: Virulence. 2017 Sep 21;9(1):133–48. doi: 10.1080/21505594.2017.1366408 (PMC5955454; doi:10.1080/21505594.2017.1366408)
Supplement: KVIR_S_1366408.zip [file kvir-09-01-1366408-s001.zip › KVIR_S_1366408_Figs.pptx]

## Slide 1
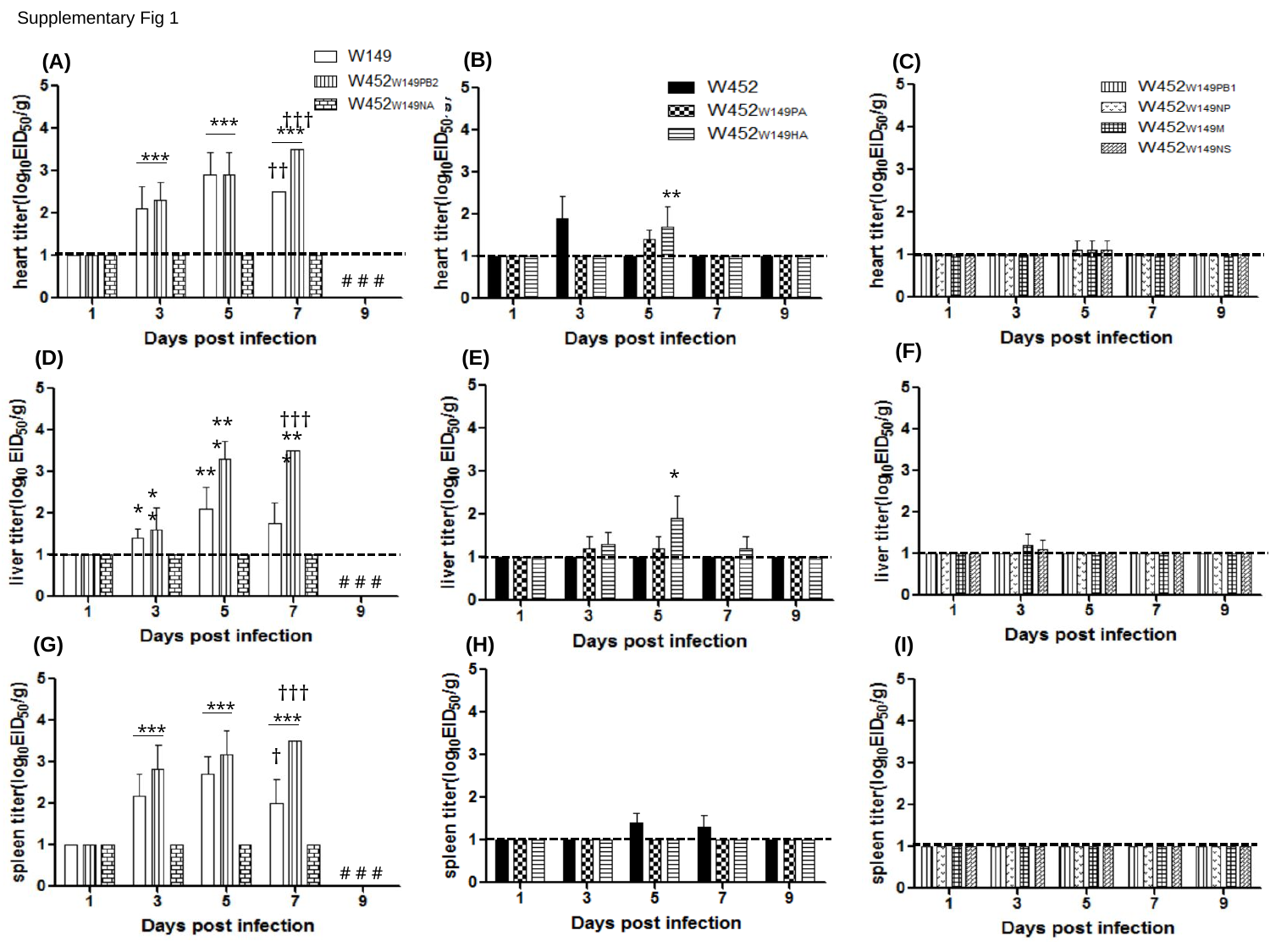

Supplementary Fig 1
(B)
(A)
(C)
†††
***
***
***
††
**
# # #
(F)
(D)
(E)
†††
***
***
**
*
**
*
# # #
(G)
(H)
(I)
# # #
†††
***
***
***
†

## Slide 2
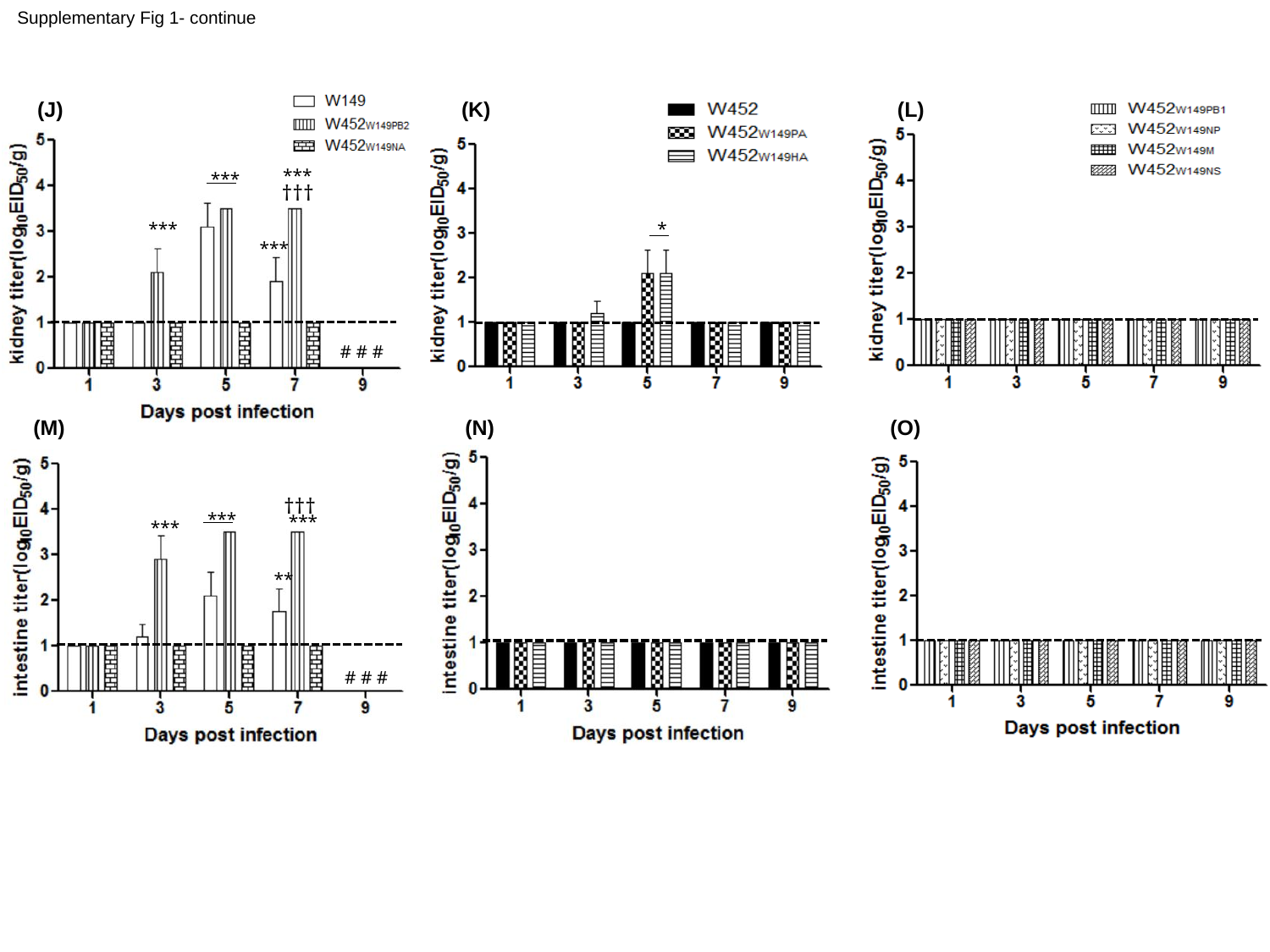

Supplementary Fig 1- continue
(J)
(K)
*
(L)
# # #
***
***
†††
***
***
(M)
(N)
(O)
# # #
†††
***
***
***
**

## Slide 3
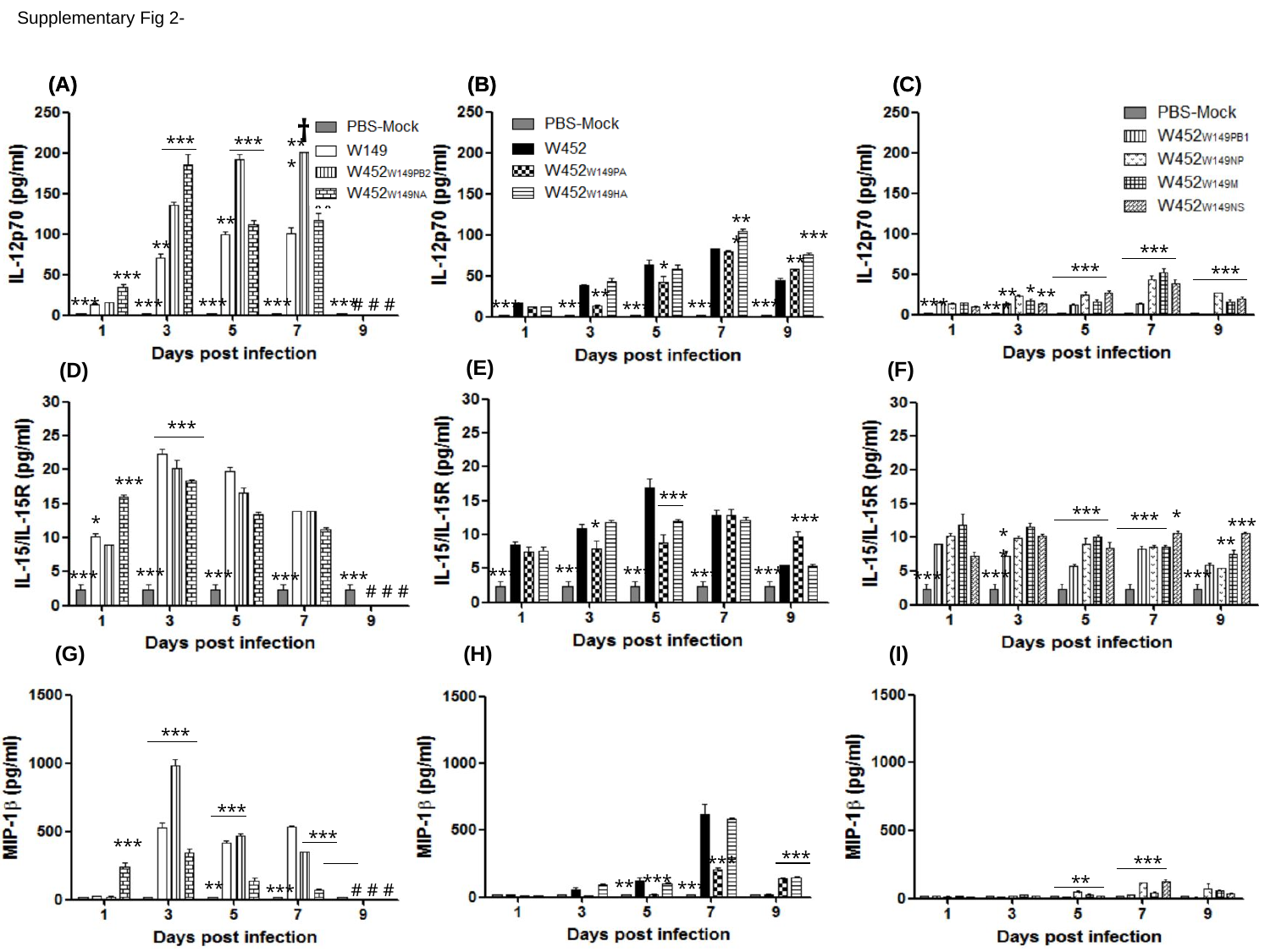

Supplementary Fig 2-
(A)
(B)
(C)
(A)
(B)
(C)
***
***
**
*
**
***
***
***
***
***
***
***
***
**
**
**
***
# # #
†
***
***
***
***
***
***
***
***
*
**
**
***
***
(E)
(F)
(D)
***
***
*
# # #
***
***
***
***
***
# # #
***
***
*
***
***
***
***
***
***
**
***
*
***
**
***
***
***
(G)
(H)
(I)
***
***
***
# # #
***
**
***
***
***
***
**
***
***
**

## Slide 4
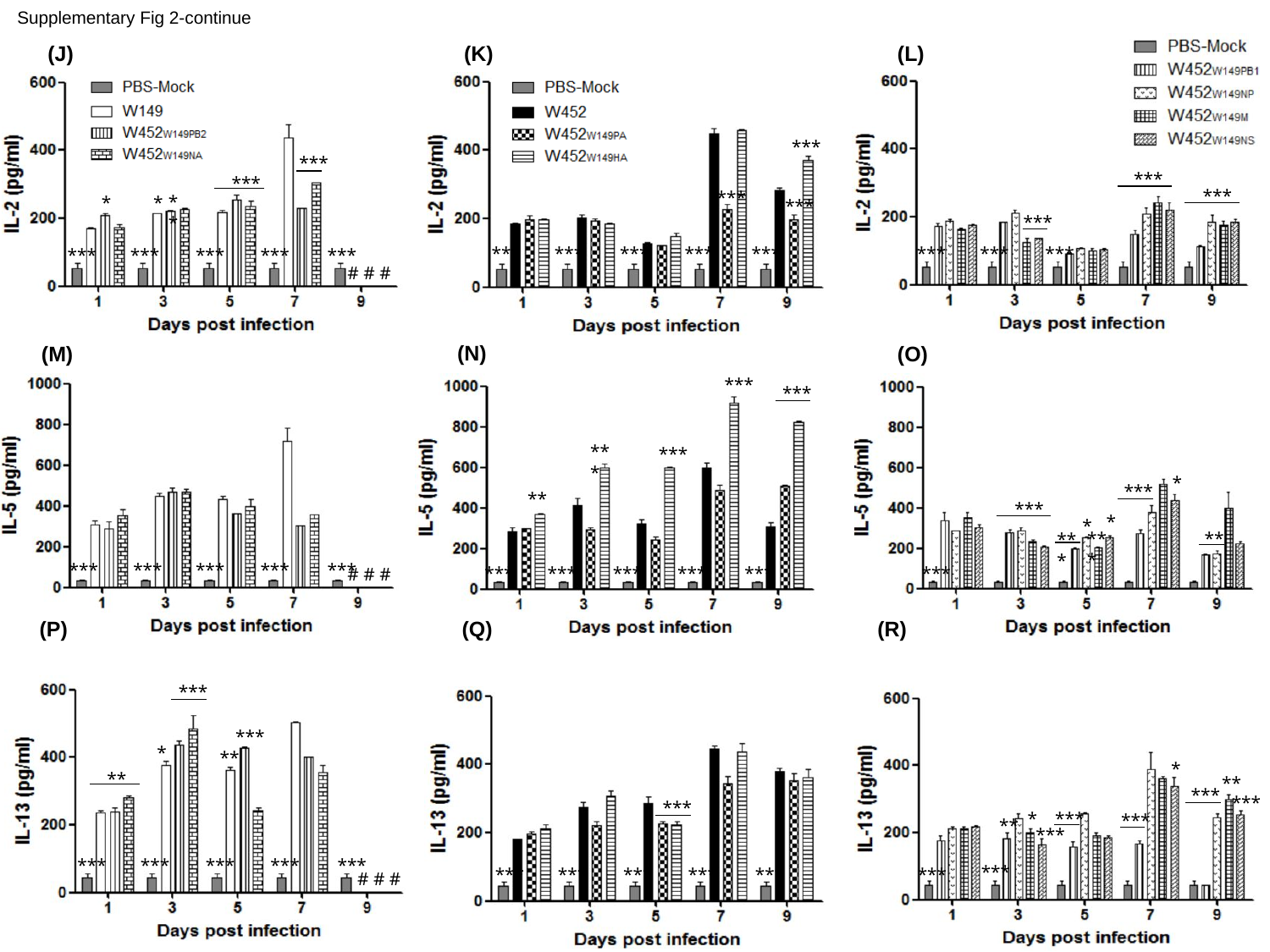

Supplementary Fig 2-continue
(J)
(K)
(L)
***
**
*
*
# # #
***
***
***
***
***
***
***
***
***
***
***
***
***
***
***
***
***
***
***
***
(N)
(M)
(O)
***
***
***
***
**
***
***
***
***
***
***
***
***
***
***
# # #
*
***
***
*
*
***
**
***
***
(P)
(Q)
(R)
***
***
*
**
**
# # #
***
***
***
***
***
*
**
***
***
*
***
***
**
***
***
***
***
***
***
***
***
***

## Slide 5
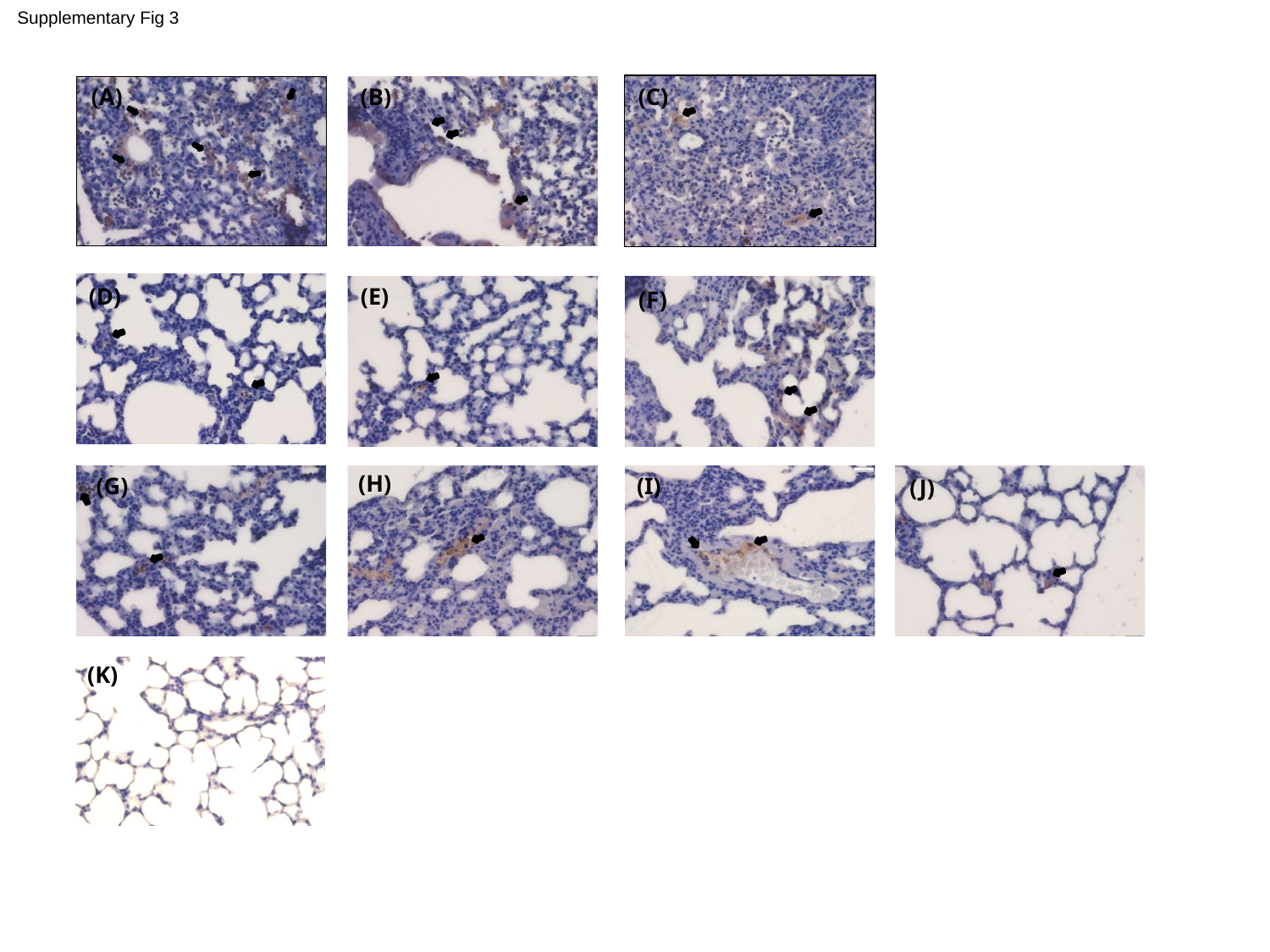

Supplementary Fig 3
(A)
(B)
(C)
(D)
(E)
(F)
(H)
(I)
(G)
(J)
(K)

## Slide 6
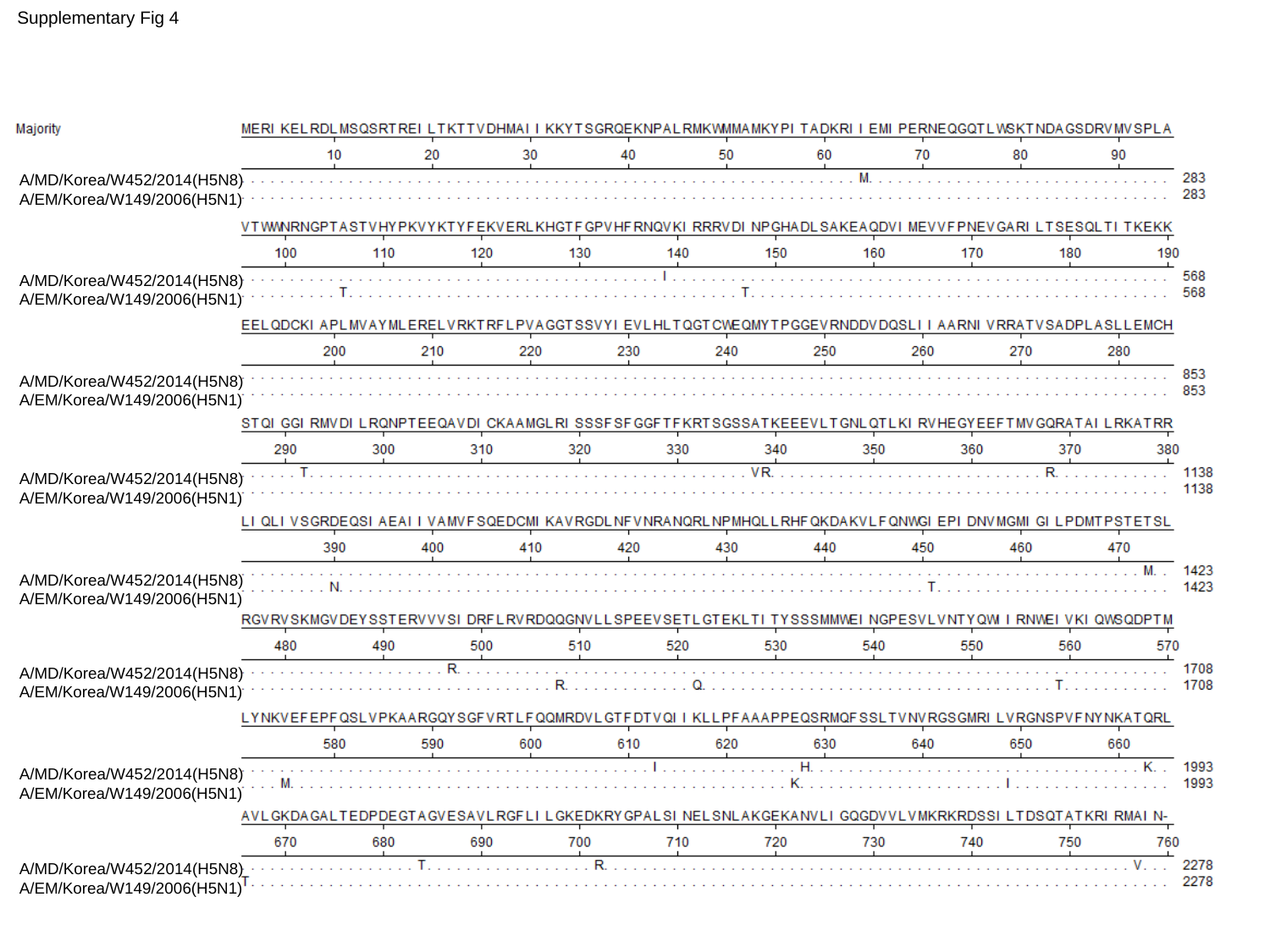

Supplementary Fig 4
A/MD/Korea/W452/2014(H5N8)
A/EM/Korea/W149/2006(H5N1)
A/MD/Korea/W452/2014(H5N8)
A/EM/Korea/W149/2006(H5N1)
A/MD/Korea/W452/2014(H5N8)
A/EM/Korea/W149/2006(H5N1)
A/MD/Korea/W452/2014(H5N8)
A/EM/Korea/W149/2006(H5N1)
A/MD/Korea/W452/2014(H5N8)
A/EM/Korea/W149/2006(H5N1)
A/MD/Korea/W452/2014(H5N8)
A/EM/Korea/W149/2006(H5N1)
A/MD/Korea/W452/2014(H5N8)
A/EM/Korea/W149/2006(H5N1)
A/MD/Korea/W452/2014(H5N8)
A/EM/Korea/W149/2006(H5N1)
